# A Semiautomated Classification System for Producing Service Directories in Social and Health Care (DESDE-AND): Maturity Assessment Study

**DOI:** 10.2196/24930

**Published:** 2021-03-15

**Authors:** Cristina Romero-Lopez-Alberca, Federico Alonso-Trujillo, Jose Luis Almenara-Abellan, Jose A Salinas-Perez, Mencia R Gutierrez-Colosia, Juan-Luis Gonzalez-Caballero, Sandra Pinzon Pulido, Luis Salvador-Carulla

**Affiliations:** 1 Department of Psychology Universidad de Cádiz Cádiz Spain; 2 Centro de Investigación Biomédica en Red de Salud Mental (CIBERSAM) Instituto de Salud Carlos III Madrid Spain; 3 Agencia de Servicios Sociales y Dependencia de Andalucía Junta de Andalucía Sevilla Spain; 4 Health Information Systems Group (SICA-CTS-553) Universidad de Cádiz Cádiz Spain; 5 Hospital Universitario Reina Sofía Servicio Andaluz de Salud Córdoba Spain; 6 Department of Quantitative Methods Universidad Loyola Andalucía Sevilla Spain; 7 Centre for Mental Health Research, Research School of Population Health ANU College of Health and Medicine Australian National University Canberra Australia; 8 Department of Psychology Universidad Loyola Andalucía Sevilla Spain; 9 Departamento Estadística e Investigación Operativa Universidad de Cádiz Cádiz Spain; 10 Escuela Andaluza de Salud Pública Gobierno Regional de la Junta de Andalucía Granada Spain

**Keywords:** DESDE-LTC, DESDE-AND, services coding, service directories, decision support system, impact analysis, maturity

## Abstract

**Background:**

DESDE-LTC (Description and Evaluation of Services and DirectoriEs for Long-Term Care) is an international classification system that allows standardized coding and comparisons between different territories and care sectors, such as health and social care, in defined geographic areas. We adapted DESDE-LTC into a computer tool (DESDE-AND) for compiling a directory of care services in Andalucia, Spain.

**Objective:**

The aim of this study was to evaluate the maturity of DESDE-AND. A secondary objective of this study is to show the practicality of a new combined set of standard evaluation tools for measuring the maturity of health technology products.

**Methods:**

A system for semiautomated coding of service provision has been co-designed. A panel of 23 domain experts and a group of 68 end users participated in its maturity assessment that included its technology readiness level (TRL), usability, validity, adoption (Adoption Impact Ladder [AIL]), and overall degree of maturity [implementation maturity model [IMM]). We piloted the prototype in an urban environment (Seville, Spain).

**Results:**

The prototype was demonstrated in an operational environment (TRL 7). Sixty-eight different care services were coded, generating fact sheets for each service and its geolocation map. The observed agreement was 90%, with moderate reliability. The tool was partially adopted by the regional government of Andalucia (Spain), reaching a level 5 in adoption (AIL) and a level 4 in maturity (IMM) and is ready for full implementation.

**Conclusions:**

DESDE-AND is a usable and manageable system for coding and compiling service directories and it can be used as a core module of decision support systems to guide planning in complex cross-sectoral areas such as combined social and health care.

## Introduction

COVID-19 has dramatically exposed the cracks of otherwise highly connected systems such as social and health services. For example, nursing homes are both health-related organizations and social housing facilities for highly vulnerable people [1]. This connection was mostly hidden to service planners until the patients’ and staff flows between hospitals and nursing homes during COVID-19 unleashed “a perfect storm” around the health and social care systems locally and globally [2]. This pandemic has also shown the relevance of computer modeling, care navigation systems, real-time dashboards, and interactive decision support systems to guide evidence-informed policy. However, to be effective, rapid response digital systems should go beyond traditional semantic interoperability across data sets, and move toward a common coding and counting of service provision (availability, bed capacity, and workforce) from a whole system’s perspective (health, social, housing, employment, education, and justice) [3-5]. This harmonization effort should follow a service ecosystem approach [6] and focus on the comprehensive assessment of the overall local and regional service availability and capacity as it has also been underscored by the pandemic [1]. The World Health Organization (WHO) Advisory Committee on Health Research has pointed out to the importance of developing useful policies based on evidence of the availability of services in catchment areas [7,8]. This cross-sectorial coding system of service provision should also allow international comparison of social and health systems, as this is essential to detect possible gaps in service capacity and provision and to conduct comparative effectiveness studies.

However, the aggregation and comparison of services from a whole system’s perspective is not an easy task, as shown by previous developments in disability or functional dependency in Europe [9,10], older adults [11], and mental health and chronic care [12]. These attempts have been recently extended to education [3] and justice [4] where similar problems have been identified.

The difficulty of comparability between services across sectors is related to a number of factors including [13-15] (1) the geographic variability of care systems; (2) the noncommensurability bias or lack of consensus on the units of analysis for comparisons at macro (countries, regions), meso (small health areas), and micro (individual services) levels [16]; (3) the terminological variability of the services and programs, and the lack of a workable formal ontology of service provision in the main health terminology systems such as SNOMED; (4) and the low usability and comparability of official directories or listings of services available at the regional or national level. These factors highlight the importance of a consensus on the taxonomy and classification of services analogous to that existing for the classification of diseases, classification of operations, and classification of health interventions (eg, in the World Health Organization Family of International Classifications [17]); and the importance of using instruments for service assessment with published psychometric data that may facilitate disambiguation and enhance semantic interoperability in service assessment and monitoring.

DESDE-LTC (Description and Evaluation of Services and DirectoriEs for Long-Term Care) is a standardized coding system for care services to be used across sectors and tools such as health and social directories, atlases of care, and decision support systems [10,12,18,19]. This system identifies and codes basic care teams (named Basic Stable Inputs of Care [BSIC]) as its main unit of analysis and applies a code to their main activity called “Main Type of Care.” It provides local bottom–up multisectoral coding [20] of care services across different target groups and has been tested in mental health care [21,22], intellectual disability [23], alcohol and drug abuse [24,25], general disability [10], aging, and chronic or long-term care [16,26]. DESDE-LTC has been translated into 9 languages and applied in over 34 countries [27]. The information gathered using this classification system has proven its usefulness for designing decision support systems to guide regional mental health planning in Spain [28] and Australia [29] when combined with local information on the context of care (eg, sociodemographic data), the use of resources (eg, activities, interventions), and indicators of results [28,30].

Despite its uniqueness and tested psychometric properties, the applicability of DESDE-LTC is limited due to the significant effort in data gathering that requires surveys and direct interviews to managers of local services, as well as an intensive training for coders [15]. Mapping an urban health or social district (eg, between 300,000 and 1 million inhabitants) takes over 6 months and is a research-intensive task [31]. Therefore, an automated version of this ontology system is needed for its routinization in local and regional care planning.

This study evaluates the maturity of DESDE-AND, an online computer system to produce a semiautomatic classification of services for compiling standard directories of care based on DESDE-LTC. “Maturity” has been defined in software development as the potential capability of the process of implementation of a new product and the consistency with which it could be applied in projects throughout the target organization [32]. The capability maturity model is used in business and technology sectors for establishing life cycle and planning sustainment and has been applied to the evaluation of the process of implementation of medical products and in health care research [33]. A secondary objective of this study is to show the practicality of a new combined set of standard evaluation tools for measuring the maturity of health technology products.

## Methods

### Study Design

This study follows a co-design/hybrid approach. The co-design process was based on information provided by a domain expert panel and key end users. The initial expert panel consisted of 28 professionals from different social and health areas of the regional public Agency for Social Services and Dependency of Andalucia (ASSDA). This group included experts in information technologies, policy planning, management, and service research. Training on the DESDE-LTC coding and on the use of the DESDE-AND prototype (version 0.0) was provided to all the experts. From this group, 23 domain experts participated in the panel discussion of the maturity study. The group of end users included the managers of the 68 services that participated in the demonstration study.

### Setting

The tool has been designed in a collaboration partnership between Psicost Research Association and the public ASSDA. Within the regional Ministry of Equity and Social Policies of Andalucia (Spain), ASSDA coordinates the social public and private care and the welfare system for the 8.5 million inhabitants living in this Spanish region [34]. The regional government has full governance on planning, funding, and managing its health and social care systems. Apart from social services, the Ministry of Equity and Social Policies regulates and manages a complex mix of health and social services for specific target groups (eg, drug and alcohol, aging, disabilities) as well as other support services such as housing, special employment, and special education services. It also participates in the interministerial agency of mental health involving the departments of health and social services. The Social Services Law [35] has made mandatory the development of the Social Services Map of Andalucia, and recommends the implementation of a “classification system for the different types of services for planning and evaluation.” The tool has been tested in the city of Seville (target area), with nearly 700,000 inhabitants.

### Instruments

The DESDE-LTC is a tool developed from the European Service Mapping Schedule [36,37]. This instrument presents a hierarchical tree structure with 6 main service branches, at 6 levels of granularity, and its last version includes 124 possible codes to describe the typology of each service assessed. The tool is distributed into 4 sections. Section A includes the general principles of evaluation and coding, the description of the reference area, and the target population of the services to be codified in the assessment process. Section B consists of a tree that represents the hierarchical structure, the description of the codes, and their identification by means of labels of the main health care types. Section C collects the use of services (Basic Stable Inputs of Care) in a reference area. Finally, section D includes a service inventory that collects data on the main characteristics of each service.

### Implementation Maturity Model (IMM)

The implementation maturity model (IMM) uses the 5 maturity levels from the capability maturity model to assess and determine the degree of maturity of implementation processes in software engineering [32,38]. In Level 1 (Initial), the organizational capability process is not structured, and is undefined and inconsistent; it largely relies on successes of individuals. In Level 2 (Repeatable), the process has partially consistent, successful processes; and Level 3 (Defined) has standard and documented processes. In Level 4 (Managed), the process is quantitatively managed, while the process is Optimized in Level 5 [33]. From these definitions, the level of maturity of the different aspects of the implementation process can be confirmed, thus describing their strengths and weaknesses, and also where improvements are needed. This ordinal rating is designed for self-assessment of the process of development of a product.

### Technology Readiness Level (TRL)

Readiness is the level of preparedness for the application of a new scientific knowledge for commercialization or generalized use in the real world. The technology readiness levels (TRLs) are a systematic measurement that supports assessments of the maturity of a particular technology during the acquisition phase of a program. Nine levels are considered [39]: TRL 1, Basic principles observed and reported; TRL 2, Technology concept and/or application formulated; TRL 3, Analytical and experimental critical function and/or characteristic proof of concept; TRL 4, Component and/or breadboard validation in laboratory environment; TRL 5, Component and/or breadboard validation in relevant environment; TRL 6, System/subsystem model or prototype demonstration in a relevant environment; TRL 7, System prototype demonstration in a space environment; TRL 8, Actual system completed and “flight qualified” through test and demonstration; TRL 9, Actual system “flight proven” through successful mission operations.

### Psicost Usability Checklist

The Usability Checklist consists of 15 items that assess relevance (meaningfulness, novelty, and potentiality of the new scientific knowledge to the target audience), acceptability (the degree to which a new application or product is agreed or approved by the target audience), functionality, security, practicality (related to implementation, training requirements, and complexity of evaluation, and the analysis, interpretation, and reporting of the data), efficiency (practicality in relation to effort, time, and costs), training, and agreement. It uses a 10-point Likert scale, in which 0 is none, and 10 is the maximum score of each assessed variable. The questionnaire can be adapted to the characteristics of any new tool and the target end users. It has been used in the assessment of decision support systems [30] and was adapted for the assessment of the DESDE-AND in the target organization [15]. The usability questionnaire allows the study of the feasibility of a tool in its initial version, as well as the analysis of its final usability after its pilot study.

### Adoption Impact Ladder (AIL)

The Adoption Impact Ladder (AIL) is an inventory for evaluating the level to which the target organization has taken the new knowledge or its application as its own. It uses a quasi-ordinal scale with 7 categories, namely, (0) no adoption; (1) awareness; (2) assimilation; (3) conversion (or translation); (4) allocation; (5) provision; and (6) routinization (or monitoring). It has been previously used in Australia for assessing the impact on the practice of a state policy program (Ed-LinQ Program in Queensland) [40], the international dissemination of a classification of case management [41], the adoption of the Spanish Disability Scheme by all the regional agencies in Andalucia [42], and in the use of DESDE-LTC for mapping mental health services in Spain [19]. Its validation study in Spain has indicated high usability and good reliability for assessing the impact of the adoption of multisectoral social and health policies and plans [42].

### Procedure

**Figure 1:**
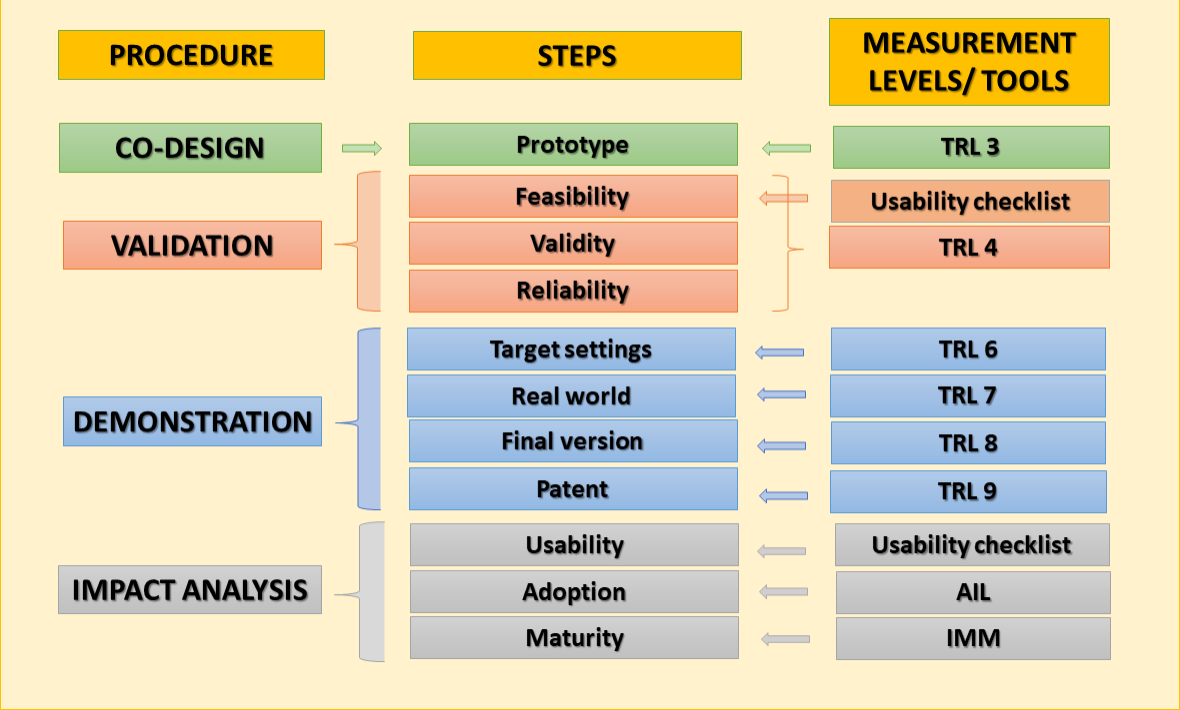
Stages of the DESDE-AND study methodology. AIL: Adoption Impact Ladder; IMM: implementation maturity model; TRL: technology readiness level.

This study consists of 4 stages comprising 11 steps and 4 measurement instruments (Figure 1).

#### Stage 1: Co-design of the DESDE-AND Prototype

The tool was built as an online computer system that guides “end users” step by step. The end user of this computer tool was defined as the manager or other decision maker of a service depending on the regional Ministry of Equality, Social Policies and Conciliation of Andalucia.

The development of the computer tool used architectural standards and was specifically programmed using JAVA. The software developer company (Guadaltech S.L.) prepared the user/administrator manuals, installation guides, implementation manual, and source code.

Two additional computer modules were developed to explore the combined use of DESDE-AND as a core module of a DSS (a data export system linked to geographic information system [GIS] for visualization and spatial analysis) and to assess its usability.

#### Stage 2: Validation of the DESDE-AND Prototype (Version 0.0)

The validation process of the DESDE-AND was part of level 4 of the technology readiness. A mini-Delphi panel of 23 domain experts produced the expert’s knowledge base [43] on the DESDE-AND prototype. During the proof-of-concept phase, the experts conducted an assessment of the feasibility of the prototype (ie, the usability during the development process). The Psicost Usability Checklist was used to assess the feasibility prototype after coding a set of case examples from the real world. We calculated the averages of the scores of the different domains of feasibility: relevance, acceptability, functionality, security, practicality, efficiency, and agreement. The interrater reliability was analyzed during the demonstration phase. Kappa coefficients were calculated by comparing the coding made by an expert in DESDE-LTC (alpha evaluator) and the 68 managers of the services selected for the demonstration phase (beta evaluators). Finally, we analyzed the predictive validity of the codes obtained using DESDE-AND.

#### Stage 3: Demonstration Study—DESDE-AND Version 0.1

This stage included a pilot study and the assessment of the DESDE-AND practicability and manageability. The pilot study was carried out in the Seville metropolitan area. A total of 188 services providing care authorized from the regional Ministry of Equality, Social Policies and Conciliation of Andalucia were identified in the target area and invited to participate in the study. This list included 13 services located outside the target area that provided types of care that were not available in the city of Seville but that could be used by their residents. As much as 73 services accepted to participate but 5 of them were not able to complete the information requested. Finally, 68 services participated in the demonstration study.

In order to perform the coding, the managers of the services (end users) identified the basic social and health care units of each service and automatically assigned them a “main type of care” code according to the decision tree system offered. This code was subsequently confirmed by the personnel responsible for public administration.

The panel of experts carried out the analysis of the results of the coding in the selected services, which allowed the preparation of a report with recommendations for improvement of the instrument.

The recommendations by the panel of experts allowed modifications and improvements to be made for the development of the instrument in its final version (version 1.0).

#### Stage 4: Impact Analysis

The Psicost Usability Checklist, which was first used to assess the feasibility of the prototype by the 23 domain experts, was completed at the end of the demonstration phase by the 68 end users to assess the usability of the tool. The adoption by the target organization was measured by 3 experts in service evaluation and in the use of the decision support tools (FA-T, MG-C, and JS-P). The level of adoption was assessed in the individual services and in the target organization using AIL.

Every stage of the development process of the tool corresponded to a TRL. At the completion of the final version (DESDE-AND version 1.0), the group of domain experts assessed the level of technological maturity reached by the tool (TRL), which was compared with the one provided by the core team. Finally, the overall level of maturity was assessed using the IMM.

## Results

### Stage 1: Co-Design of the DESDE-AND Prototype

**Figure 2:**
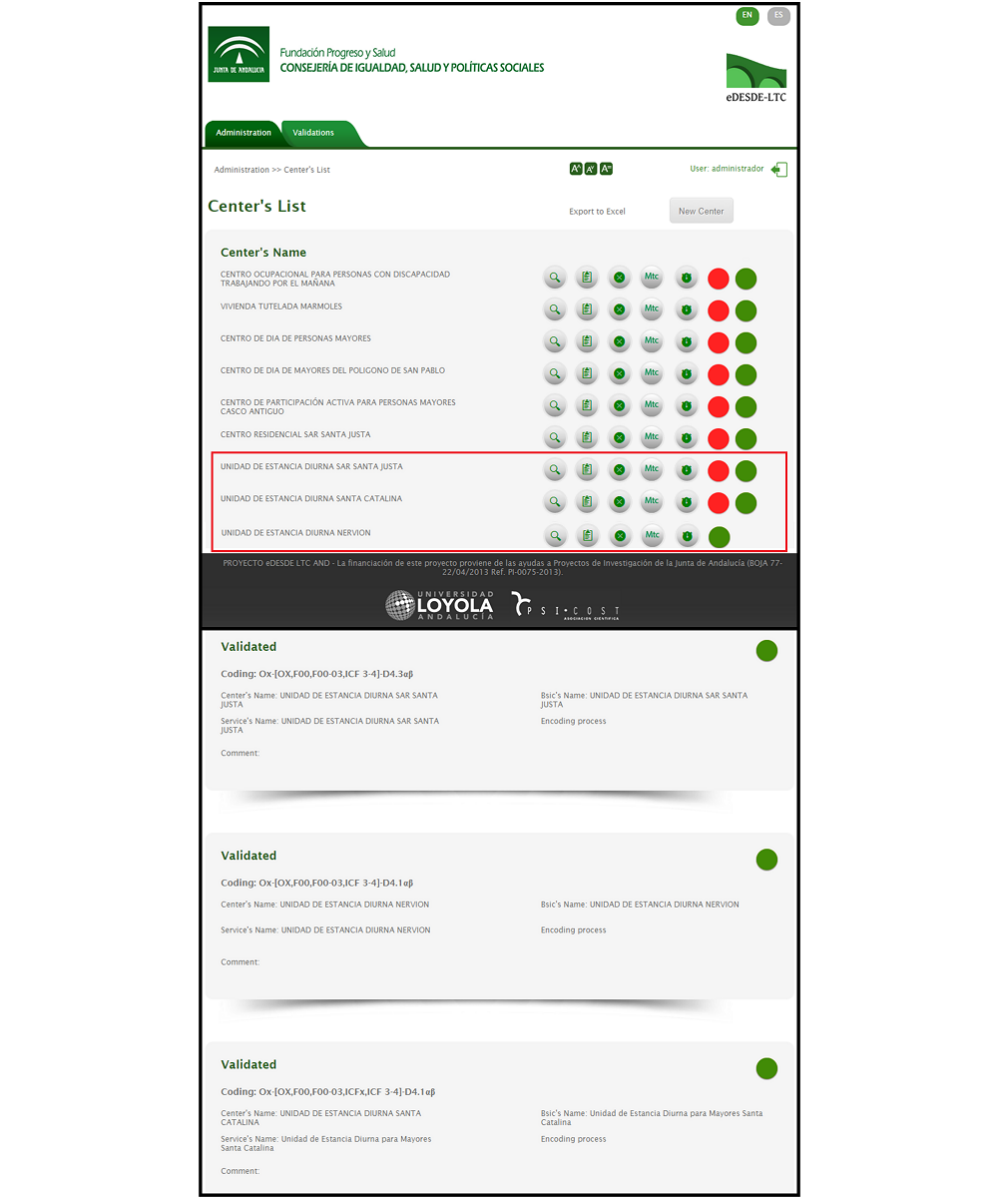
User interface of DESDE-AND web application.

The schemas and algorithms necessary for the development of the computer-administered version 0.0 (prototype) were designed in the first stage. The classification process used specific question answering algorithms with 2 or more logical answers in a decision tree. Subsequently, a user interface for the web application was created with a user-friendly design, using a set of images and graphic objects to represent the available actions (Figure 2).

A modular system with minimal interconnection between modules was used to develop the software and user interfaces.

The modular operability of DESDE-AND was tested with a supplementary module designed for exporting georeferenced information from the coding generated in the service inventory to any GIS and online cartographic viewers. This has been used to demonstrate the capacity of DESDE-AND data to be exported to other modules within a composite decision support system. GIS is the most common visualization system in DSS [44]. It allows performing complex spatial analyses, such as context studies (sociodemographic situation of the geographic environment of the services), assessing the optimal location of services and their accessibility. The results of the pilot study were integrated into the open GIS of Google Maps.

### Stage 2: Validation of the DESDE-AND Prototype (Version 0.0)

**Figure 3:**
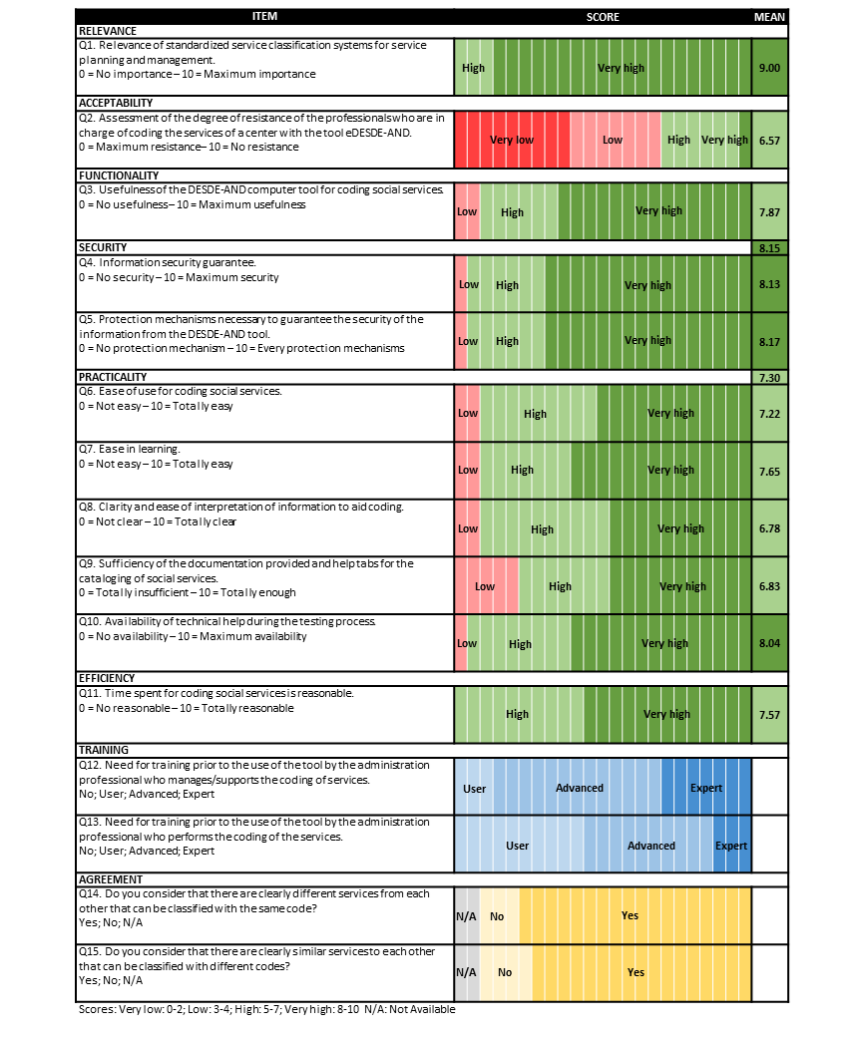
Experts’ responses to the usability questionnaire. Each cell represents the qualitative score or answer of a different expert, and the last column shows the mean of their quantitative score by question and domain.

The feasibility of the tool was assessed using the Usability Checklist. A total of 23 domain experts completed the instrument (Figure 3). The “Relevance” items were rated high (average score of 9). The “Acceptability” was relatively low, with an average score of 6.57. Questions about “Functionality” obtained an average score of 7.87. The items that assessed the “Practicality” of the tool obtained an average rating of 7.3, while “Efficiency” reached an average score of 7.57. Regarding the need for prior training, 80% (19/23) of the experts considered that the 68 service managers should have prior training at an advanced level, and 57% (13/23) of the experts considered that the advance training level should be also provided to the personnel that classified the services. The experts had an average agreement of 76.1%.

The interrater reliability analysis was performed after the pilot study in the 68 services. We assessed the coding concordance between ratings provided by the 68 service managers and the experts. The agreement was 0.89 with a Kappa index of 0.33 (95% CI). We also calculated the predictive validity of the tool (sensitivity, specificity, and predictive values), and observed high results in specificity (89.39%; 95% CI 81.21-97.58), sensitivity (100%), and predictive value (100%).

### Stage 3: Demonstration Study—DESDE-AND Version 0.1

The pilot study included 68 services that agreed to participate and were classified using the DESDE-AND. The remaining services did not respond to the request, because they were closed or because they did not have adequate human or technical resources. Six of them started the process but did not complete the coding because they did not have personnel with basic training in information technology and communication. The 68 coded services were designed for different target groups: child and adolescents (n=5, 7%), older adults (n=40, 59%), persons with disabilities (n=16, 24%), drug addiction problems (n=5, 7%), and community social services (n=2, 3%).

The assessment of usability of the final version of DESDE-AND was performed by the service managers for the 68 services of the pilot study. Average scores were 7.6 for relevance, 7.8 for functionality, 7.8 for usability, 6.9 for efficiency, and 2.4 for resistance of the staff to perform the coding. More than half of the of the professionals (13/23, 57%) who coded the services considered that training was necessary at the user level in order to use the DESDE-AND.

We received 63 improvement suggestions and comments on the prototype to produce version 0.1. Of these, 52 recommendations were accepted and, among them, the corrections considered were performed to ensure gender equality. Eleven proposals were discarded due to technical issues, programming, or errors in the use of the tool.

**Figure 4:**
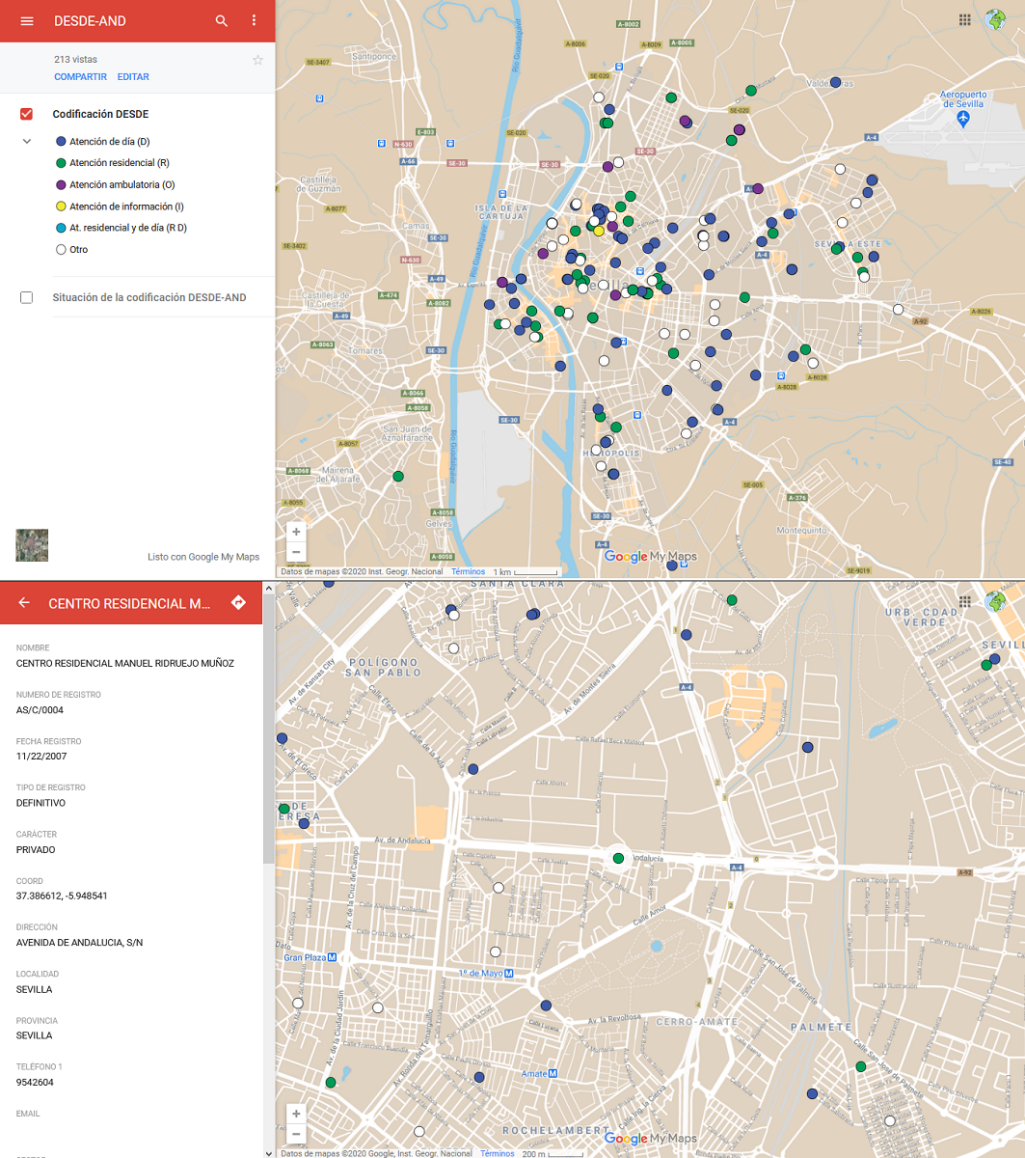
Screenshots of the web-mapping application (Google Maps) for the geographic visualisation of the services coded through DESDE-AND tool for social care planning in Andalucia (Spain).

The results of the pilot study in Seville were integrated into the open GIS of Google Maps [45], allowing the geolocation of the results of DESDE in any standard GIS system in the market. Figure 4 provides a depiction of the actual digital tool: the online directory of services, the classification using the DESDE coding system, and the geolocation of the services in the catchment area. This figure shows the importance of disambiguation in social care before they are geolocated. There are services that are officially identified with the same name that receive 2 different DESDE codes and are identified as different type in the GIS.

### Stage 4: Impact Analysis

The level of adoption was assessed in the individual services registered for the study as well as in the main target organization (ASSDA) by 3 experts (FA-T, MRG-C, and JAS-P). According to these experts, DESDE-AND reached an AIL level of 5 (impact on the budget, financing, and allocation of resources for testing the tool in the target environment without routine use within the organization).

The overall maturity level of the tool was moderate-high or Managed (IMM 4): the process of implementing is documented throughout the organization rather than per aspect, and projects are carried out under the guidance of project operation standards and an implementation strategy [38].

This process has generated a computer tool with a semiautomated core module to obtain the standard directory of the health and social services available in a given area, based on an international classification.

## Discussion

### Principal Findings

It uses a multiaxial system that allows typify entities into differentiated groups using a string of codes or “axes” corresponding to the main attributes of the entity from other groupings in the same system [46]. This multiaxial system classifies the services according to the geographical catchment area, the target population, and the type of care provided (residential care, day care, outpatient care, information, accessibility, or self-help). It includes a file with detailed information relating to each of the available services reducing disambiguation and enhancing the semantic interoperability of health and social care databases. The improvement of the applicability and practicality ratings of DESDE-AND in comparison with the ones obtained by the paper and pencil version [15] indicates a better usability, although the actual time for completion and updating the coding and mapping of care districts will require a further analysis after routinization.

This classification can be used by managers with the expert support provided by the computer tool, simplifying the complex task of coding in real time, and unifying the taxonomic and semantic criteria. It has been developed using a co-design approach focused on end users (managers, planners, and other decision makers) [47,48]. It ensures that the design satisfies the users of the system by making improvements in the usability and human performance, thus reducing previous training requirements in length and intensity.

DESDE-AND is a system that allows various functions and different levels of web surfing/navigation and use: (1) a simple easy navigation for consumers; (2) a second advanced level of web surfing for case managers to improve care continuity; and (3) an advanced level that can be incorporated to decision support tools for social and health planning. This tool facilitates the analysis of health care ecosystems [6], covering the various sectors, levels, and types of services involved in the care provision of an area, as well as the local characteristics and the drivers of the system (socioeconomic, demographic, legislative, and political). This information is relevant for recognizing and understanding the pattern of care provision, care gaps, unmet needs, and duplications of any system [20].

This system has been developed using a co-design approach. Co-design is largely used to improve service quality and delivery [49], and can also be used for the purpose of policy making [50,51]. Using co-design to inform policy making is particularly important to mental health care as we recognize that co-creation of well-being is critical and principal actors are vulnerable [48,52]. Our study uses co-design to improve the quality of indicators by accounting for contextual and environmental factors as well as to foster knowledge transfer of decision and policy making prior to policy design. By doing so, the system can afford to inform and bridge the resource gaps between the quality of service provision and the quality of decision and policy.

The original instrument DESDE-LTC has demonstrated its use in national and international comparisons of service directories and atlases of health and social care [27]. As DESDE-AND uses the same coding system, it will improve the comparisons across jurisdictions. This is key for service research, traditionally hampered by intrinsic problems in assessments of services [13]. This tool also facilitates the identification of complex patterns of provision and differences in national and international health and care research and monitoring [16,22,53]. It could improve the analysis of associations between the level of availability of services and sentinel indicators such as deprivation or social fragmentation [54], suicide rates [55], family burden [56], employment [57], or the cost of illnesses [58].

The information related to the coding of services can be exported to a GIS. This modular function facilitates data filtering by regions or provinces, municipalities, sectors of care, and types of services. It enables rapid geo-positioning of services with operational identification criteria and their specific characteristics. Henceforth, DESDE-AND facilitates the development of social and health care atlases of care [19]. Atlases are one of the visual tools currently being used for the assessment and planning of health systems [44].

The basic information obtained also allows the development of service indicators classified by geographic study areas, such as rates of services availability per inhabitants, beds (placement capacity), and professionals (workforce capacity); the analysis of the diversity of care in an area; and the balance between health and social care [30]. It could also be used to analyze accessibility and optimal locations of services. Atlases using the DESDE system have been made available in Spain [59], Europe [22,60], Australia [31,61], and Chile [53].

The final implementation of the tool in Andalusia depends on a series of regulatory steps. The Andalusian Social Services Law of 2017 entails the Map of Social Services of Andalusia and its update. The DESDE coding system will be included in the next version of the map.

The Map of Social Services of Andalucia is in operation. It is a computerized map of social services that allows the geolocation of the centers and services in the Catalogue of Social Services of Andalucia [62]. The inclusion of a field with an international reference code for the standardized classification of each center typology, such as DESDE-AND, constitutes an example of possible practical applications of the tool in planning social services in a certain area.

Finally, and as a secondary result, this study illustrates the use of standard measures and instruments for the quantitative assessment of maturity, a key phase in implementation research.

### Limitations

First, this computer tool uses an internet-based algorithm adapted to the specific conditions of the Autonomous Community of Andalucia, and to the information data sets of the Andalusian Social Services. The transferability to other territories has not been tested. However, the study describes the process and tools required to produce similar semiautomated tools adapted to other environments. Second, there is a lack of international agreement and guidelines for conducting impact analysis of digital health care [63] and the domains of maturity that require evaluation. We opted for the overall ordinal assessment of the maturity level (IMM [38]), complemented by the ordinal rating of key domains of maturity instead of conducting in-depth interviews to stakeholders. “Maturity” is considered here as part of the “early implementation” phase [64].

Although the maturity assessment instruments used in this study were developed for self-rating and monitoring within organizations, they can provide useful information on the calibration of impact analysis across different projects, areas, and sectors in health technology assessment. We used a mixed approach combining evidence and expert knowledge, and by applying a co-design process [65] selected a defined catchment area using a health care ecosystem approach [20,66,67], and identified the target organizations in this catchment area. The maturity general rating [33] and the maturity domains selected in this study are supported by previous literature in health care technology assessment: the “TRL” (adopted by the European Commission and Horizon programs) [39]; the “Usability” of the Tool in a real world environment [15]; and its “Adoption” by target organizations, as suggested by Health Quality Ontario [66].

### Conclusions

DESDE-AND is a usable and manageable computer tool for guiding evidence-informed decision making in health and social planning. This tool reduces ambiguity and increases semantic interoperability in health and social care. It improves a previous tool that has demonstrated its usability for detecting gaps and inequities in the provision of health and social care. DESDE-AND is a relevant contribution to establish a common terminology, classification, and coding of health and social care services in the national context, as well as a standardized procedure for data collections and comparisons.

## Abbreviations

AIL: Adoption Impact Ladder
DESDE-LTC: Description and Evaluation of Services and DirectoriEs for Long-Term Care
IMM: implementation maturity model
TRL: technology readiness level
